# Vertical semiconductor deep ultraviolet light emitting diodes on a nanowire-assisted aluminum nitride buffer layer

**DOI:** 10.1038/s41598-022-11246-0

**Published:** 2022-05-04

**Authors:** Qihua Zhang, Heemal Parimoo, Eli Martel, Songrui Zhao

**Affiliations:** Department of Electrical and Computer Engineering, McGill University, 3480 University Street, Montreal, Quebec H3A 0E9 Canada

**Keywords:** Lasers, LEDs and light sources, Lasers, LEDs and light sources

## Abstract

Vertical light-emitting diodes (LEDs) have many advantages such as uniform current injection, excellent scalability of the chip size, and simple packaging process. Hitherto, however, technologically important semiconductor aluminum gallium nitride (AlGaN) deep ultraviolet (UV) LEDs are mainly through lateral injection. Herein, we demonstrate a new and practical path for vertical AlGaN deep UV LEDs, which exploits a thin AlN buffer layer formed on a nanowire-based template on silicon (Si). Such a buffer layer enables in situ formation of vertical AlGaN deep UV LEDs on Si. Near Lambertian emission pattern is measured from the top surface. The decent reflectivity of Si in the deep UV range makes such a configuration a viable low-cost solution for vertical AlGaN deep UV LEDs. More importantly, the use of such a thin AlN buffer layer can allow an easy transfer of device structures to other carrier wafers for vertical AlGaN deep UV LEDs with ultimately high electrical and optical performance.

## Introduction

Deep UV light sources play a critical role in our everyday life for a wide range of applications in disinfection, UV curing in the production of any personal electronic devices, and bio-chemical sensing. Today, the dominant technologies have remained relying on mercury lamps, which are hazards to both the environment and human health. In this context, a significant effort has been devoted to the development of semiconductor deep UV LEDs based on AlGaN alloys, which are the materials of choice for semiconductor deep UV LEDs^1–12^.

In general, there are two schemes for the current injection of an LED device: vertical and lateral. Comparing to the lateral scheme, the vertical scheme offers a number of advantages such as uniform current injection, excellent scalability of the chip size, and simple packaging process^6,13–16^. A uniform current injection is also critical for laser devices^17,18^. Nonetheless, realizing vertical AlGaN deep UV LEDs remains to be a challenge in the field; and the majority device demonstrations of AlGaN deep UV LEDs so far are through lateral current injection. The latest review on this topic can be found, e.g., in Ref.^6^.

Two common approaches of fabricating vertical LEDs are: (1) using conductive substrates, and (2) substrate removal and bonding to a second carrier wafer. Both approaches, however, are difficult to implement for vertical AlGaN deep UV LEDs. Today, AlGaN deep UV LEDs are mainly on insulating sapphire substrates, precluding the in situ vertical current injection. On the other hand, although n-SiC and n-GaN substrates are conductive, they have a number of limitations. The lattice mismatch between GaN and AlN is a known challenge^6^, whereas although the lattice mismatch between SiC (6H) and AlN is small, it faces a substrate cost penalty^19^. More adversely, both n-SiC and n-GaN have a strong deep UV light absorption. As such, in both scenarios, i.e., insulating substrates (sapphire) and conductive substrates (n-GaN, n-SiC), substrate removal is necessary.

Laser lift-off (LLO) has been successful in the fabrication of InGaN visible color LEDs since the first demonstrations around 2000^13,14^. However, the success cannot be transferred to AlGaN deep UV LEDs, due to the need of AlN buffer layers for AlGaN deep UV LEDs. The LLO of AlN is difficult due to the high melting point of AlN, as well as the generation of Al during the LLO process, which can lead to cracks and is difficult to remove^20–25^. This is in addition to a possible device degradation incurred in the LLO process^26,27^.

Different from substrates mentioned above, Si substrate can be removed easily by wet etching processes^28,29^. Moreover, Si substrate is available at a large size at a low cost and is thus favorable for mass production. However, growing high quality AlGaN epilayers on Si is a challenge. The large tensile stress in AlN and high-Al content AlGaN alloys, due to their large lattice mismatches with Si (e.g., 19% for AlN)^30,31^, leads to poor material quality (e.g., cracks, poor surface morphology). To mitigate the challenge, various approaches, such as low-temperature (LT)/high-temperature (HT) AlN buffer layers, epitaxial lateral overgrowth (ELO)-AlN buffer layers, AlGaN superlattices (SLs), and graded AlGaN buffer layers, have been developed in order to obtain high quality AlGaN device layers^30–36^. These approaches, however, require the use of complicated and time-consuming substrate patterning processes or growth processes. Moreover, buffer layers of several μm thick are required in order to obtain high quality device layers. The thick, insulating buffer layers used in these approaches only led to laterally injected AlGaN deep UV LEDs^30,33,36,37^.

An alternative path for vertical AlGaN deep UV LEDs is to use nanowire structures^38,39^. However, the fabrication of AlGaN nanowire deep UV LEDs is one remaining issue. This is mainly due to the presence of gaps amongst nanowires. For example, due to the presence of gaps, certain planarization is required. Conventionally, this is done by polymer backfill. However, the commonly available polymers absorb deep UV light strongly and degrade under deep UV light illumination. It is ideal to have an AlGaN deep UV LED technology on Si that combines the advantage of nanowires (e.g., better stress relaxation) and epilayers (e.g., manufacturing-compatible device fabrication process).

Recently, we have shown that by using a nanowire template, AlN epilayers can be formed on Si, which have further led to AlGaN epilayers with a relatively high internal quantum efficiency^40–42^. In this work, we further demonstrate vertical AlGaN deep UV LEDs on Si following the idea of nanowire-assisted AlN buffer layer but with modifications: First, to improve the quality of the nanowire template, a pre-nanowire AlN layer is introduced to remove any residual tensile stress on the nanowire template from the Si substrate. Second, metal-rich condition, rather than nitrogen-rich condition, is used for the growth of the AlN buffer layer, in order to achieve a smoother surface. The use of metal-rich conditions in general promises a smoother surface compared to using N-rich conditions in a radio-frequency nitrogen-plasma-sourced molecular beam epitaxy (MBE) system. Devices emitting in the deep UV range are demonstrated using such a modified AlN buffer layer in situ on Si. As Si is a decent reflector in the deep UV range^43,44^ and highly electrical and thermal conductive, such a configuration could be a possible path of fabricating vertical AlGaN deep UV LEDs and offers a potential benefit of direct integration to other electronic components on Si. More importantly, due to the thickness of the AlN buffer layer is very thin, it can be removed easily by chemical wet etching, which is compatible with the fabrication process of vertical InGaN visible color LEDs on Si substrates^28,29,45,46^, allowing for the achievement of vertical AlGaN deep UV LEDs with ultimately high electrical and optical performance.

## Results

**Figure 1:**
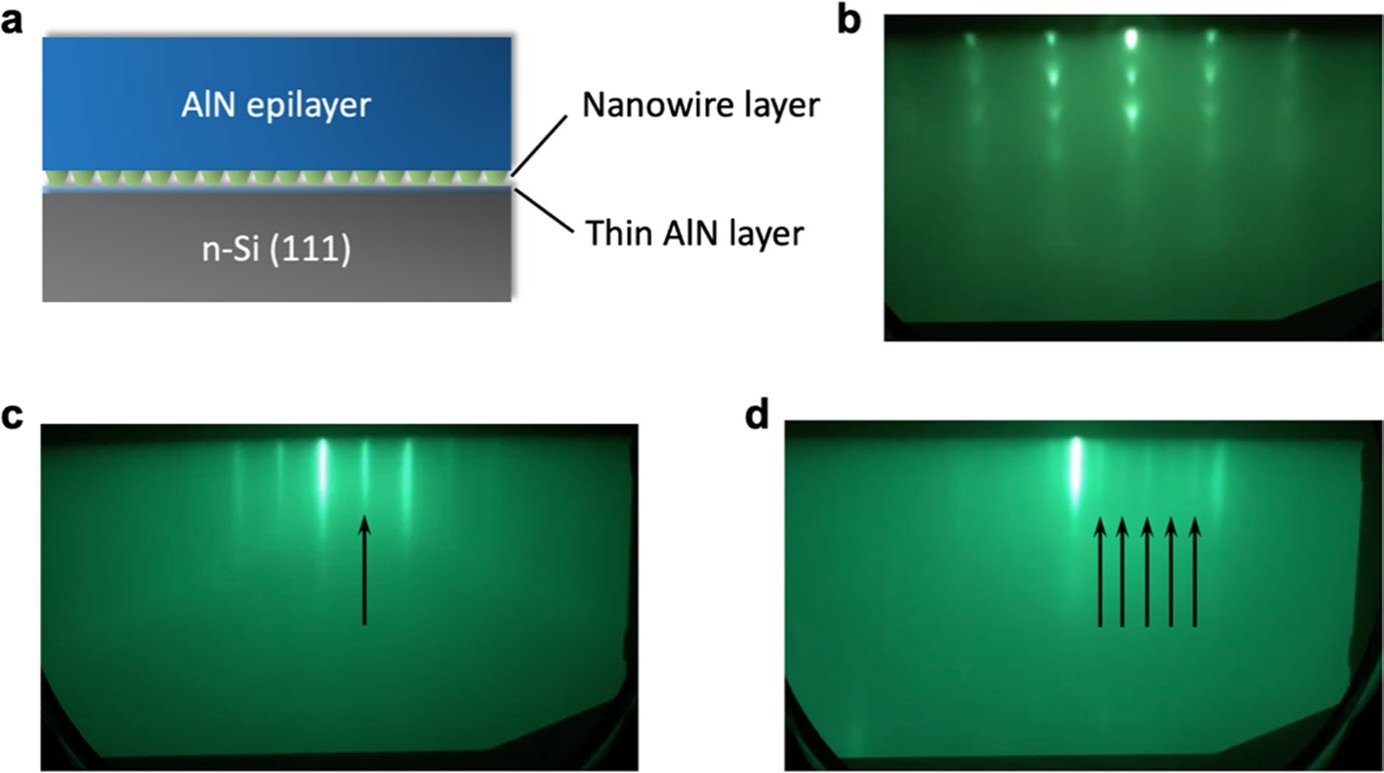
(**a**) Schematic of the growth of the AlN epilayer on Si substrate using a nanowire-based template. (**b**) RHEED pattern taken during the growth of the nanowires. (**c**, **d**) RHEED patterns taken along the < $11\overline{2}0$> direction and < $1\overline{1}00$> direction, respectively, during the growth of the AlN epilayer. The arrows reflect the 2 × 6 RHEED reconstruction.

The MBE growth and characterization of the AlN epilayer is described first. The schematic of the structure is shown in Fig. 1a, which starts with a thin (1–2 nm) AlN layer, followed by a thin (50–100 nm) GaN nanowire layer. Figure 1b shows the reflection high-energy electron diffraction (RHEED) pattern during the growth of the GaN nanowires. Regularly arranged dots are seen, suggesting a 3-dimensional (3D) growth. This RHEED feature is different from the arcs as often observed from self-organized GaN nanowires, which indicates the improvement of the nanowire vertical alignment with respect to the substrate, due to the use of the thin pre-nanowire AlN layer^47,48^.

The start of the growth of the AlN epilayer led to the RHEED pattern transition from being spotty to being streaky. The Al-rich growth condition was confirmed by the presence of excess Al through the following observations: (1) the Al shutter open and close test, wherein the close of the Al shutter led to an increase of the RHEED intensity and the opening of the Al shutter led to an intensity decrease; and (2) the observation of the RHEED 2 × 6 reconstruction (Fig. 1c, d) during the growth, which suggests the presence of an Al adlayer^49,50^. Such a RHEED pattern also reflects that the AlN epilayer is Al-polar. In addition, the narrow, bright, and streaky RHEED pattern indicates a highly smooth surface.

**Figure 2:**
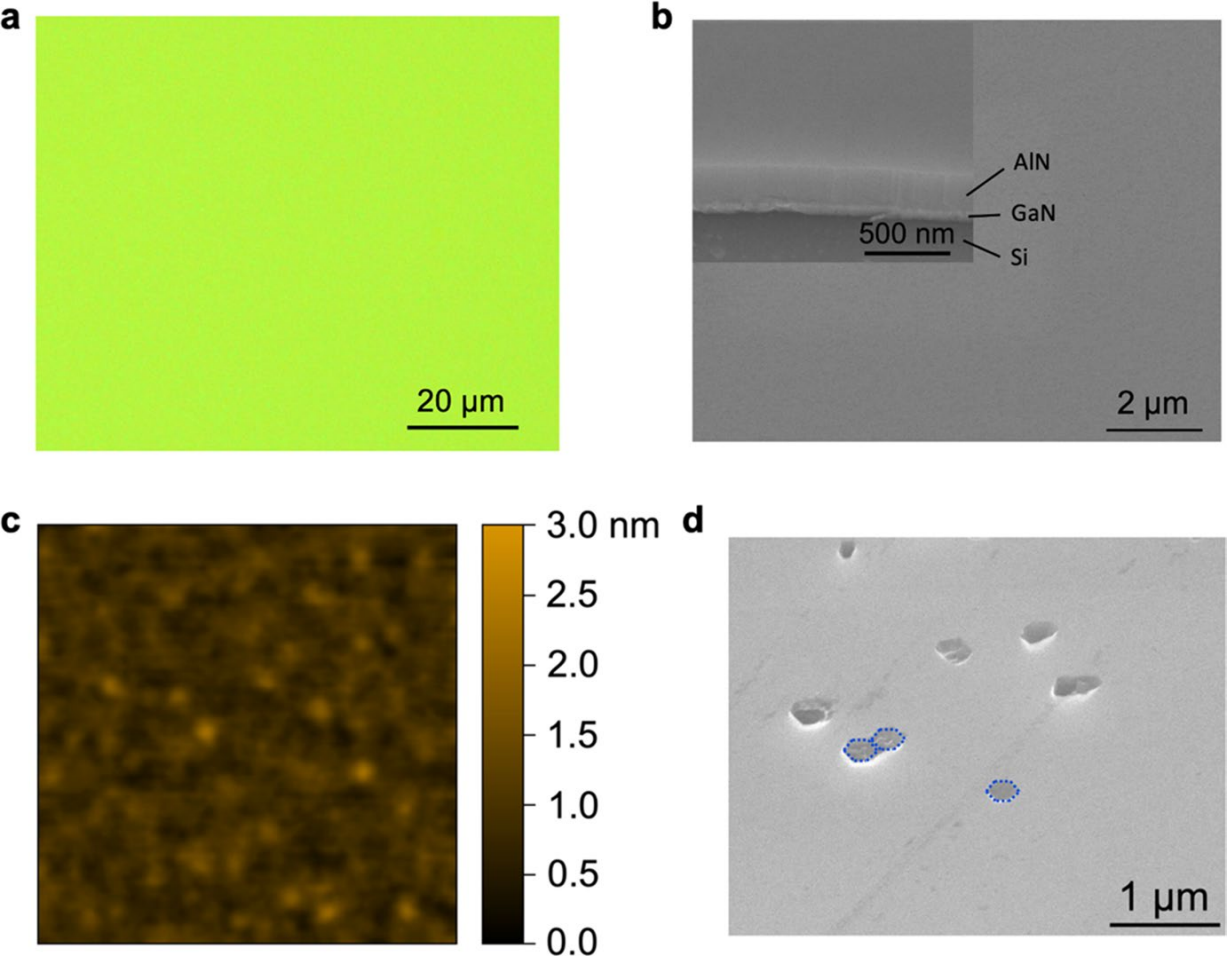
(**a**) An optical image showing the surface of the as-grown AlN epilayer. (**b**) SEM images of the as-grown AlN epilayer. Inset: An image highlighting the cross-section. (**c**) An AFM image of the surface of the as-grown AlN epilayer. The image size is 10 μm × 10 μm. (**d**) An SEM image showing the surface of the etched AlN epilayer by KOH. The dashed lines denote the hexagonal shapes and the network of hexagonal shapes.

Figure 2a shows the optical image of the as-grown AlN epilayer, an optically smooth surface can be seen. The surface of the as-grown AlN epilayer was further examined by scanning electron microscopy (SEM). The images were taken at a tilting angle of 45°. Figure 2b shows a typical SEM image, highlighting a very smooth surface. The inset of Fig. 2b shows an SEM image that manifests the cross section, with the Si substrate, GaN nanowire layer, and AlN epilayer clearly seen. The surface was further examined by atomic force microscopy (AFM). A typical AFM image is shown in Fig. 2c. For such AlN epilayers, a root-mean-square (RMS) roughness of around 0.4 nm can be obtained, which is comparable to the typical metal-polar AlGaN thin films grown on sapphire and AlN-on-sapphire template^51^. This RMS roughness is also significantly improved compared to our previous studies^41,42^.

To confirm the Al-polar polarity of the AlN epilayer, we have performed potassium hydroxide (KOH) etching experiments. In the experiments, 11.2 mol/L KOH solution was heated up to 70 °C, and the sample was entirely placed in the solution, followed by de-ionized (DI) water cleaning. The SEM image of the AlN epilayer after KOH etching is shown in Fig. 2d. It is seen that hexagonal pits, rather than hillocks, appear on the surface, confirming that the AlN epilayer is Al-polar^52–54^. This Al-polar AlN epilayer can further enable metal-polar AlGaN epilayers grown on top. The benefit is, as the opposite side of the metal-polar surface is N-polar, which can be selectively etched by KOH, it thus enables the removal of unwanted AlGaN epilayers (e.g., additional AlGaN buffer layers for material quality improvement), as well as the roughening of the surface through which the light comes out (for flip-chip devices). Indeed, the AlGaN epilayers to be described below are metal-polar as confirmed by KOH experiments.

**Figure 3:**
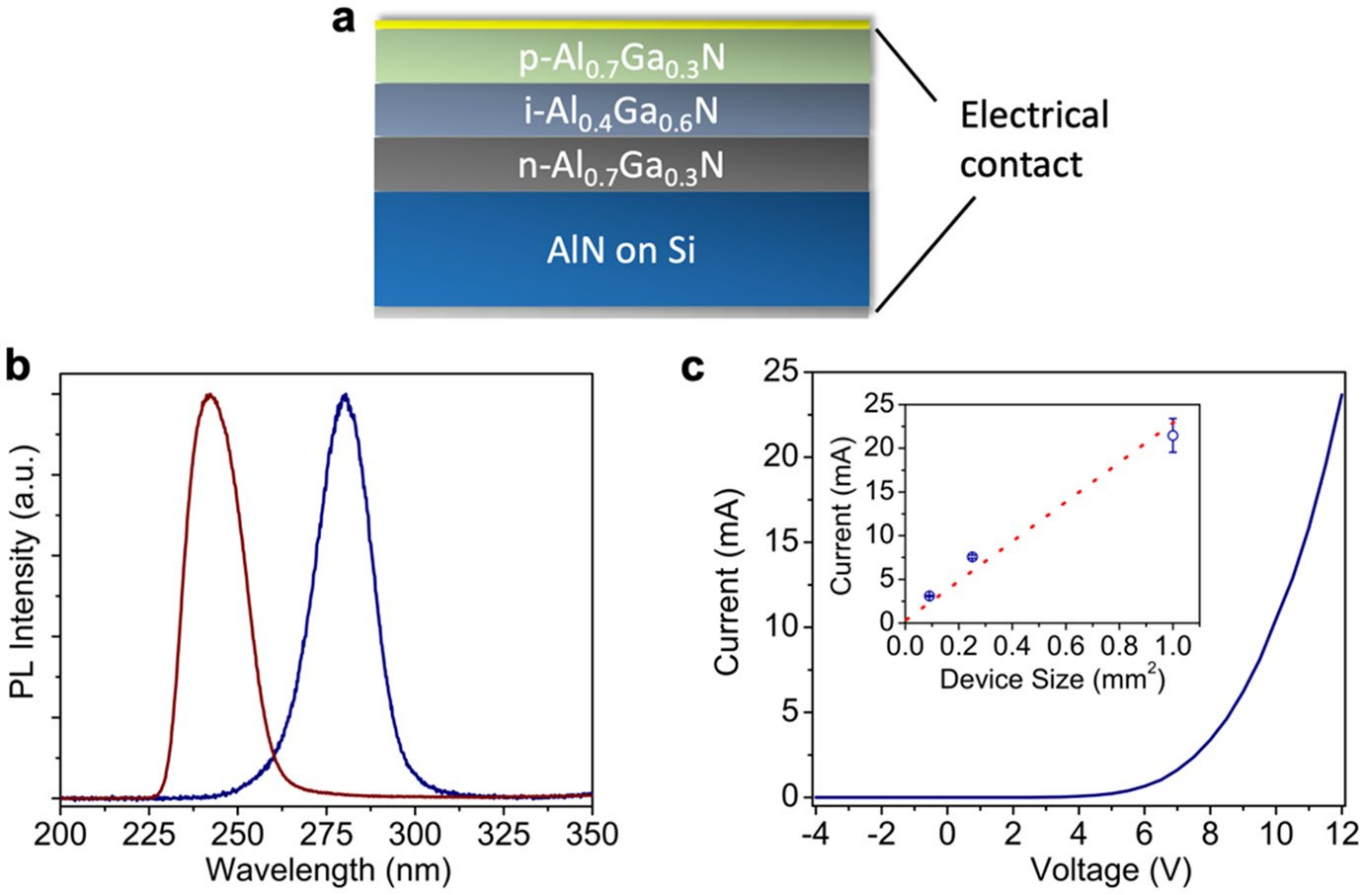
(**a**) Schematic of the AlGaN DH LED structure grown on top of the AlN buffer layer on Si. The electrical contacts are also shown. (**b**) Room-temperature PL spectra of the AlGaN active region and cladding layers. (**c**) I–V characteristics of an AlGaN DH LED with a device size of 1 mm × 1 mm. The inset shows the measured forward currents from devices with various sizes at a forward voltage of 12 V. Numerous devices were tested, and the error bars reflect the current variation from devices with the same size.

We further demonstrate vertical AlGaN deep UV LEDs using such AlN epilayers as buffer layers on Si. The schematic of the device structure is shown in Fig. 3a, which consists of double heterojunctions (DHs) with an i-Al_0.4_Ga_0.6_N active region (~ 15 nm) and *p*- and *n*-Al_0.7_Ga_0.3_N cladding layers (~ 30 nm each). These Al contents were estimated from the room temperature photoluminescence (PL) experiments from AlGaN epilayers grown separately on such AlN buffer layers. Figure 3b shows the room temperature PL spectra measured from the AlGaN epilayers intended to be used as the active region and cladding layers for the devices. It is seen that PL emission at around 240 nm and 280 nm are measured. Assuming the PL peak energy approximately to be the bandgap energy and the bowing factor to be 1^55^, the Al contents in the active region and cladding layers were estimated to be ~ 40% and ~ 70%, respectively. It is also noted that only a single PL emission peak is seen for both the active region and cladding layers, suggesting a uniform alloy composition.

For device fabrication, we did not use chemical etching to isolate devices with different sizes. The isolation was obtained by the limitation of the current spreading length in the vertical injection scheme. Our calculation indicates that under an injection current density of 0.1 A/cm^2^, with the best reported p-AlGaN resistivity (Al content of 70%) and the largest ideality factor^56–58^, as well as the present p-AlGaN layer thickness, the maximum current spreading length is on the order of tens of μm. Therefore, by placing *p*-contact with a separation on the order of several hundred μm, devices can be naturally isolated.

The room-temperature I–V characteristics under a continuous-wave (CW) biasing are shown in Fig. 3c. At forward voltages of 9 V and 12 V, the forward currents were 6 mA and 23 mA, respectively, which are improved compared to the previously reported laterally injected AlGaN thin film UV LEDs on Si at a similar operating wavelength, e.g., Ref.^33^. Moreover, the device size dependent current further suggests a uniform current injection. Illustrated in the inset of Fig. 3c is a device size dependent current under a forward voltage of 12 V, and it is seen that the current increase is proportional to the device size increase, indicating a uniform current injection.

**Figure 4:**
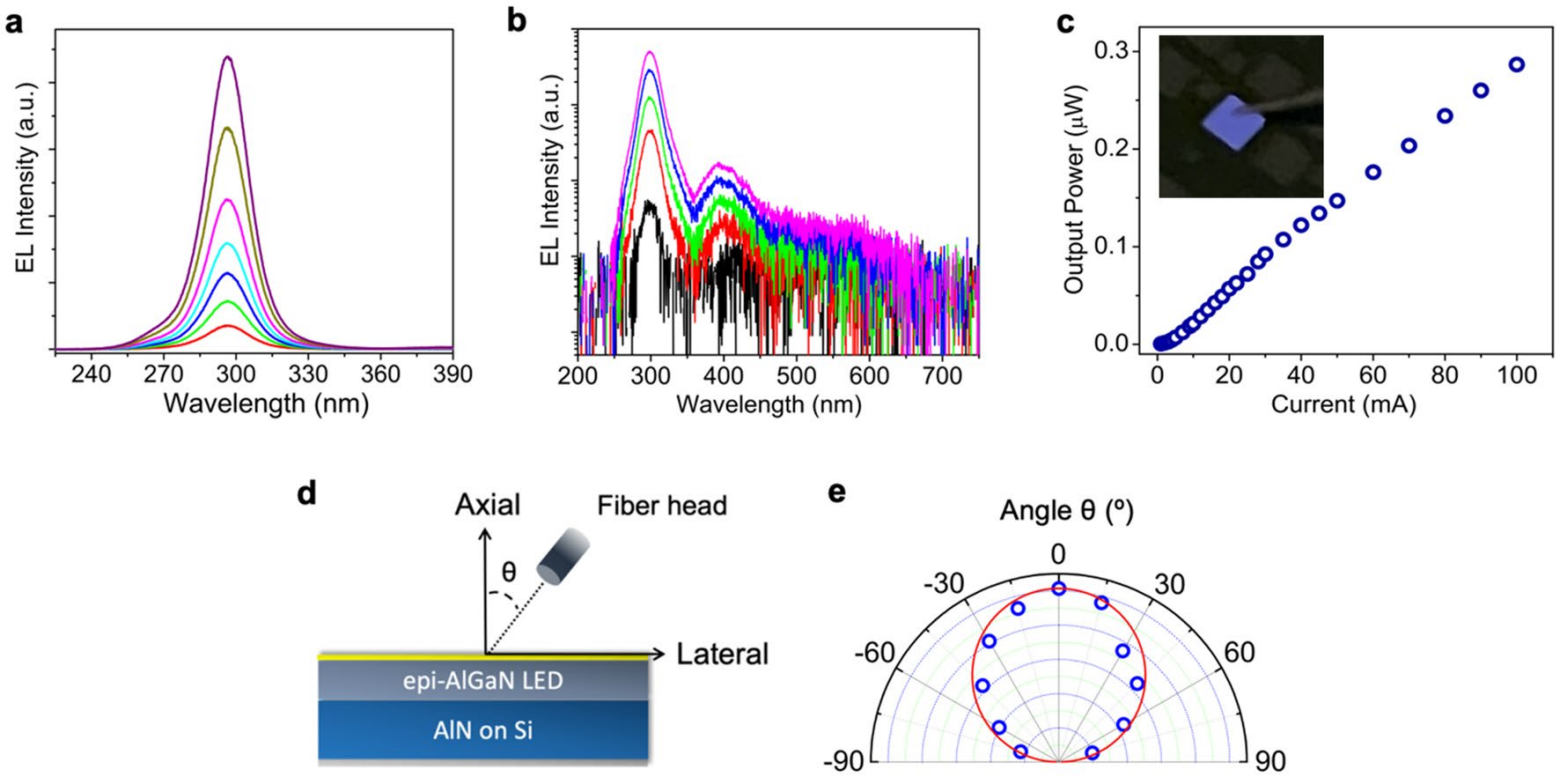
(**a**) Room-temperature EL spectra of an AlGaN DH LED under different injection currents. (**b**) Room-temperature EL spectra taken up to the visible spectral range with currents varying from 1 to 12 mA. (**c**) Light output power versus the injected current. The inset shows an optical image of the light emission. (**d**) Schematic of the setup for the angle dependent EL measurement. (**e**) Emission pattern with the detection angle θ varying from − 75° to 75°. The solid curve denotes the ideal Lambertian pattern. Device size: 1 mm × 1 mm.

The electroluminescence (EL) spectra under different injection currents are shown in Fig. 4a. It is seen that the EL emission occurs at 298 nm. With the change of the injection current from 2 to 20 mA, no noticeable EL emission peak blueshift and full-width-at-half-maximum narrowing are observed. The EL emission wavelength is longer than what we expect, and the reason is being investigated. Figure 4b shows the EL spectra measured with a UV–VIS spectrometer, intended to study the defect EL emission. A parasitic EL emission peak at around 400 nm is seen, which could be originated from the deep levels in AlGaN alloys^59,60^. Its intensity, however, is significantly lower compared to the main EL emission peak. Figure 4c shows the light output power as a function of the injection current under a CW biasing. A typical LED behavior is seen, i.e., the light output power increases nearly linearly with the injection current. Under an injection current of 100 mA, a light output power of 0.3 μW was measured. An optical image of the light emission is shown in the inset of Fig. 4c, wherein a bright and uniform emission can be seen.

The emission pattern of such LEDs is also studied. In this regard, the optical fiber was tilted at various angles with respect to the axial direction (the growth direction) for the light detection, as illustrated in Fig. 4d. The emission pattern is shown in Fig. 4e by open circles. The ideal Lambertian emission pattern is also shown by the solid curve. It is seen that the device shows a near Lambertian pattern, suggesting the nature of surface emission.

## Discussion

In this work, we have demonstrated vertical AlGaN deep UV LEDs on Si. Such devices are made on a special AlN buffer layer that is formed with the assistance of a nanowire-based template. As Si is a decent reflector in the deep UV range, such vertical devices offer a low-cost solution for vertical semiconductor deep UV LEDs and a potential benefit of in situ integration to other electronics on Si, and are suitable for low-power applications. Further improvement on the electrical performance for such devices is expected by optimizing the electrical doping; and further improvement on the light output power can be expected by optimizing the p-contact and adopting more complicated device designs such as using quantum wells and electron blocking layers. More attractively, as the thickness of the AlN buffer layer is very thin, it can be removed easily by chemical wet etching (same for the nanowires, as the sidewall of nanowires grown by MBE is N-polar, e.g., Ref.^61^), which allows the transfer of device structures grown on top to other carrier wafers for the achievement of vertical AlGaN deep UV LEDs with ultimately high electrical and optical performance. Therefore, this work enables a practical path for high performance vertical semiconductor deep UV LEDs.

## Methods

### Molecular beam epitaxial growth

All the samples in this work were grown by radio-frequency plasma-assisted molecular beam epitaxy on n-Si (111) substrates. The substrates underwent standard solvent cleaning and in situ thermal outgassing prior to the growth. The Al fluxes for the pre-nanowire AlN layer and the AlN epilayer were 2 × 10^–8^ Torr and 5 × 10^–8^ Torr, respectively. For the AlGaN DH LED structure, the Al flux was in the range of 2.8–3.5 × 10^–8^ Torr. The Ga flux was around 1.4 × 10^–7^ Torr for all layers in this study. A nitrogen flow rate of 0.6 sccm was used for the GaN nanowires, whereas for all the epilayers a nitrogen flow rate of 0.3 sccm was used. A substrate temperature in the range of 720–740 °C was used for the AlGaN epilayers; and for the AlN epilayers the substrate temperature was roughly 100 °C higher compared to AlGaN epilayers. The Mg doping concentration in the p-AlGaN layer was 1 × 10^18^ cm^−3^, estimated by the secondary-ion mass spectroscopy (SIMS, EAG lab).

### Photoluminescence experiments

A 213 nm pulsed laser with a pulse width of 7 ns was used to excite the sample. The emitted light from the sample top surface was collected by an optical fiber, which was further coupled to a deep UV spectrometer.

### Device fabrication

Metal bilayer Ni (7 nm)/Au (7 nm) was used for p-contact, which was fabricated by standard photolithography and metallization processes. The size of p-contact defines the size of the device. Colloidal Ag conductive adhesive was used on the backside of n-Si substrate as the n-contact.

### Device characterization

The electroluminescence was collected by an optical fiber from the device top surface, and both deep UV and UV–VIS spectrometers were used for the spectral analysis. The light output power was measured by a Si photodetector, which was placed roughly about 5 mm above the device top surface. The device was unpackaged.

## Data Availability

The data is available upon reasonable request to the corresponding author.
